# The Effect of Combination of Steroid and L-Asparaginase on Hyperglycemia in Children with Acute Lymphoblastic Leukemia (ALL)

**DOI:** 10.31557/APJCP.2019.20.9.2619

**Published:** 2019

**Authors:** Mururul Aisyi, Murti Andriastuti, Nia Kurniati

**Affiliations:** 1 *Department of Pediatric Hematology Oncology, Indonesian National Cancer Center “Dharmais” Cancer Hospital, *; 2 *Department of Child Health, Faculty of Medicine, Universitas Indonesia, Cipto Mangunkusumo Hospital, Jakarta, Indonesia. *

**Keywords:** Hyperglycemia, ALL induction phase, steroid, prednisone, dexamethasone, L-asparaginase

## Abstract

**Background:**

Hyperglycaemia is a common side effect of steroid and L-asparaginase combinations, occurring most often during acute lymphoblastic leukemia (ALL) induction phase. To date in Indonesia, it has not been obtained data on the incidence of hyperglycemia in children with ALL in the induction phase and how the role of combinations of L-asparaginase and different type of steroid used. The purpose of this study is to determine the incidence of hyperglycemia in children ALL induction phase, knowing the difference between prednisone and dexamethasone (in combination with L-asparaginase) in causing hyperglycemia in children with ALL and determine the relationship of other factors related to hyperglycaemia.

**Methods:**

This was a prospective analytic study with a pre- and post-test design, conducted in three hospitals (Cipto Mangunkusumo Hospital, Dharmais Cancer Hospital, and Gatot Soebroto Hospital). Patient’s blood glucose levels (BGL) were checked at the 3^rd^ (pretest), 4^th^, 5^th^ and 6^th^ week of protocol (post-test).

**Result:**

Of the 57 patients, 5.2% had hyperglycemia. The patients’ age ranged from 1.4 years old to 15.8 years old (6.7 years old). There was no relationship between age, central nervous system (CNS) infiltration, leukocytosis, Down syndrome, nutritional status, family history of diabetes, infections and ALL stratification with hyperglycemia (p>0.05). Dexamethasone has more chance of obtaining higher mean rate of change in BGL compared to prednisone. (p < 0.05; RR 10.68; CI 95% 1.52-74.73).

**Conclusion:**

The incidence of hyperglycemia in this study is 5.2%. Dexamethasone, in combination with L-asparaginase, despite having no difference in causing hyperglycemia, has an increased risk of changing BGL compared to prednisone.

## Introduction

Leukemia is a heterogeneous group of hematological malignancies which derived from abnormal or poorly differentiated hematopoietic stem cells (Miranda et al., 2019). It is responsible for 8% of all cases of cancer, including all age groups and imposes large costs regarding diagnosis and therapy (Kouhpeikar et al., 2018). Acute lymphoblastic leukemia (ALL) is the most frequent type of leukemia in children. Globally, around 1-4.75 per 100,000 children suffers from ALL, with the peak incidence rate between age 2-6 years old (Hunger et al., 2012). 

Mostert et al., (2006) reported that, out of all of the children suffering from ALL in Yogyakarta, 35% refused medication, 23% died of medication-associated causes, 22% had their conditionworsened or experienced relapse and 20% had 5-years event-free survival (EFS). Regardless of significantly better prognosis, EFS, and overall survival (OS) with risk-based stratification chemotherapy, the risk of relapse, complications during chemotherapy, and toxicity-associated death is still high (Hunger et al., 2012). Failure to receive full chemotherapy due to its toxicity has been associated with rather undesirable outcomes in children with ALL (Hijiya and van der Sluis, 2016).

**Table 1:** Demographic Data of the Subjects

Characteristics	Frequency	Percentage (%)
Age	Mean: 6,7	
	SD: 3,98	
Age		
< 10 years old	41	71.9
≥ 10 years old	16	28.1
Hospital Origin		
Cipto Mangunkusumo Hospital	33	57.9
Dharmais Cancer Hospital	14	24.6
Gatot Soebroto Hospital	10	17.5
Gender		
Male	39	68.4
Female	18	31.6
BMI Category		
Overweight	7	12.3
Normal	50	87.7
CNS Infiltration		
Yes	4	7.0
No	53	93.0
Leukocytosis (>50.000/μl)		
Yes	12	21.1
No	45	78.9
ALL Stratification		
Standard Risk	23	40.4
High Risk	34	59.6
Down Syndrome		
Yes	2	3.5
No	55	96.5
Family History of DM		
Yes	20	35.1
No	37	64.9
Infection		
Yes	22	38.6
No	35	61.4

**Table 2:** Characteristics of BGL (mg/dL) before and after Given Steroid and L-asparaginase Combination During the Induction Phase of Chemotherapy

Blood glucose level (mg/dl)				
	3^rd^ week	4^th^ week	5^th^ week	6^th^ week
Mean	90.54	94.67	90.14	105.68
SD	17.71	28.69	25.37	81.99
Median	87.00	91.00	85.00	86.00
Min-max	58-139	58-263	57-191	50-651

**Table 3:** Correlation between ALL Stratification and Hyperglycemia

	4^th^ week			6^th^ week		
	Hyperglycemia	Normoglycemia	p	Hyperglycemia	Normoglycemia	p
HR	1	34	0.614*	2	33	0.373*
SR	0	22		0	22	

*, fisher exact test; HR, high risk; SR, standard risk

**Table 4:** Correlation between Family History of DM, Leukocytosis, Nutritional Status, CNS Infiltration, Age, and Infection with the Incidence of Hyperglycemia

	4^th^ week			6^th^ week		
	Hyperglycemia	Normoglycemia	p*	Hyperglycemia	Normoglycemia	p*
Family history of DM						
Positive	1	19	0.351	0	20	0.417
Negative	0	37		2	35	
Leukocyte						
≥ 50.000/μl	0	12	0.789	0	12	0.620
< 50.000/μl	1	44		2	43	
Nutritional Status						
Overweight	0	7	0.877	1	6	0.232
Normal	1	49		1	49	
CNS Infiltration						
Positive	0	4	0.930	0	4	0.863
Negative	1	52		2	51	
Age						
≥ 10 years old	0	16	0.719	1	15	0.486
< 10 years old	1	40		1	40	
Infection Incidence						
Yes	1	21	0.386	2	20	0.145
No	0	35		0	35	

*, fisher exact test

**Table 5:** The Mean Difference between BGL before and after given the Steroid and L-asparaginase Combination Chemotherapy in the Induction Phase of ALL (1/BGL)

Variable	Pre-post	Mean difference	SD	p (CI)
Inversed blood glucose level	3^rd^ and 4^th^ week	0.00028	0.00296	0.482 (-0.00051 – 0.00106)
	3^rd^ and 5^th^ week	-0.00034259	0.00309	0.407 (-0.00116430 – 0.00047912)
	3^rd^ and 6^th^ week	0.00025019	0.00384943	0.626 (-0.0007712 – 0.00127158)

**Table 6:** The Effect of Different Kinds of Corticosteroid (in combination with L-asparaginase) to the Median Value of BGL

Variable	3^rd^ week			4^th^ week			5^th^ week			6^th^ week		
	>88	≤88	p	>92	≤92	p	>86	≤86	p	>87	≤87	p
Dexamethasone	15	20	0.598	15	20	0.847	15	20	0.598	21	14	0.038*
Prednisone	11	11		10	12		11	11		7	15	

*Data was analyzed with X^2^test; considered significant if p ≤ 0.05

**Table 7:** The Effect of Different Kinds of Corticosteroid (in combination with L-asparaginase) with the Mean Value of 3^rd^ Delta (difference between BGL in week 6 and week 3)

Variable	Mean of the 3^rd^ delta		p (CI)
	Above the mean	Below the mean	
Dexamethasone	17	18	<0.001*(1.528-74.733)
Prednisone	1	21	

*Data was analyzed with X^2^test; considered significant if p ≤ 0.05

Hyperglycemia is one of the most common side effects of steroid and L-asparaginase combination during the induction phase of chemotherapy. However, reports have variable results. Taking the incidence of hyperglycemia into consideration (the incidence being between 9,7%-56%; Lowas et al., 2009). Zhang et al., (2014) study has shown association between the incidence of hyperglycemia 5-year overall survival rate and the relapse-free survival rate. To date, there is still no official report available on the incidence rate of hyperglycemia in children with ALL receiving induction phase of chemotherapy in Indonesia. Additionally, the role of combining L-asparaginase with different kinds of steroids used in the events of hyperglycemia is still unclear. The importance of obtaining that data could not be underestimated as it is crucial to know how high or low the incidence of hyperglycemia is, and to see whether a routine check on blood glucose level (BGL)- especially in that particular group of patientsis needed or not.

## Materials and Methods

This research is an analytic prospective study with pre-post test design in order to assess the role of corticosteroid (dexamethasone and prednisone), and L-asparaginase exposure to children with phase induction ALL. The research was conducted in the Hematology-Oncology Clinic and Ward of Pediatrics at the following hospitals: Cipto Mangunkusumo Hospital, Dharmais Cancer Hospital, and Gatot Soebroto Hospital, taking time from September 2016 to June 2017. The inclusion criteria used in this research are the all ALL patients undergoing the induction phase of chemotherapy, and all patients aged 0-18 years old. Meanwhile, patients receiving high-dose and long-term steroid treatment for other diseases besides ALL, patients with hyperglycemia or previous history of diabetes mellitus, and patients whose parents or guardians are not willing to participate in this research, were excluded from the study

The minimum of sample was calculated using the estimated proportion formula. Based on these calculations, the minimum of samples needed is 52 subjcets. Samples were chosen consecutively from ALL patients undergoing the induction phase of chemotherapy following the Indonesian Protocol of Acute Lymphoblastic Leukemia 2013. The BGL of patients with ALL undergoing the induction phase of chemotherapy were checked in the third week (before they were given L-asparaginase), and once every week up to the sixth week of protocol. 

Characteristics of the subjects included the origin of the hospital, gender, age (<10 years old or >10 years old), body mass index (BMI) category (overweight or normal), CNS infiltration status (Yes or No), leukocyte level >50.000/μl (Yes or No), ALL stratification (standard risk or high risk), Down syndrome (Yes or No), family history of diabetes mellitus (Yes or No), and whether there was infection during the induction phase (Yes or No).

*Statistical Analysis*

The data were analyzed through Statistical Package for the Social Sciences (SPSS) version 21 software. A descriptive analysis of population was compiled using percentage (%) for categorical variables and mean, median, standard deviation, and maximum and minimum for continuous variables. Fisher exact test was used to determine the association of risk factors with the incidence of hyperglycemia, meanwhile to compare the proportion of post-test result from the prednisone and L-asparaginase group and the dexamethasone and L-asparaginase group were analyzed through Chi-square Test. Paired t-test was used to compare the average of ALL patients before and after chemotherapy. p-value less than 0.05 were considered statistically significant. The dependent variable of this study is hyperglycaemia, while the independent variable is combination between steroid and L-asparaginase.

## Results

As many as 57 patients from three hospitals were collected. Most of them are from Cipto Mangunkusumo Hospital 57.9% patients. The mean age of the patients was 6.7 years (SD = 3.98). Table 1 presents demographic data of the patients. The majority of patients were < 10 years old (71.9%), from Cipto Mangunkusumo Hospital (57.9%), male (68.4%), and BMI Category normal (87.7%). 93% of the patients No CNS Infiltration, 23,1% have leukocyte >50.000/μl, and 59.6% high risk ALL stratification. Characteristics of BGL before and after given steroid and L-asparaginase was present in Table 2. The median of BGL in the 4th week was 91.00 (58-263), meanwhile median the 6th week was 86.00 (50-651). 

There is no correlation between ALL stratification with hyperglycemia on both 4^th^ week and 6^th^ week. On the 4^th^ week p value was 0.614 and on the 6^th^ week p value was 0.373. In Table 4, it can be seen that there is no correlation between all the risk factors with incidence of hyperglycemia on both weeks. The result of paired t-test to compare the mean difference between before and after given the steroid and L-asparaginase. Statistically, there is no mean difference between BGL before and after given the steroid and L-asparaginase on 3^rd^ week and 4^th^ week, 3^rd^ week and 5^th^ week, and 3^rd ^week and 6^th^ week. 

From the calculation of relative risk based on the median on the 4th measurement BGL, it was concluded that dexamethasone administration was not a protective factor nor risk factors for median BGL above 87 compared to prednisone. For the difference between BGL pre and post-test in each week (delta BGL pre-post), a result of 15,14 were obtained for the 3^rd^ delta (6^th^ week BGL minus 3^rd^ week BGL). A recode was then conducted based on the mean of 15. With an *X*^2^ test, analysis of the influence of different kinds of corticosteroid (dexamethasone and prednisone) towards the mean of 3^rd^ delta was performed (see Table 7). Results showed that, from the relative risk count based on the 3^rd^ delta, administering dexamethasone results in 10,68 times the chance of getting an above-average score for the difference in BGL compared to prednisone. This result is statistically significant with a confidence level between 1,528-74,733.

## Discussion

In our research, the age interval of the patients is between 1,4 years old up to 15,8 years old, with a mean value of 6.7 years old. Most of the patients come from Greater Jakarta (76.4%) and male patients are more common than female patients (68.4% vs 31.6%). Patients were taken from three different hospitals in Jakarta; Cipto Mangunkusumo Hospital (57.9%), Dharmais Cancer Hospital (24.6%), and Gatot Soebroto Hospital (17.5%).

The incidence of hyperglycemia in this research is 3/57 (5,26%). This value is lower compared to previous research, such as that of Pui et al., (1981) (9,7%), Baillargeon et al., (2005) (11%), Robertson et al., (2008) (16%), Lowas et al., (2009) (20.4%), Sonabend et al., (2009) (34%), and Koltin et al., (2012) (15.6%). This is due to the age of the patients in our research, who are mostly below 10 years of age (71.9%), with normal BMI value (87.7%), and no history of diabetes mellitus in the family (64.9%). These three factors are most often associated with the incidence of hyperglycemia.

Our research did not find a correlation between overweight and the incidence of hyperglycemia. This result may be due to the significantly lower amount of overweight patients as compared to patients with normal weight in our research (12.3% and 87.7%, respectively). On the contrary, Sonabend et al., (2008) had a high amount of patients with obesity (36%), and therefore reported a high prevalence of hyperglycemia in the induction phase.

The measurements of BGL did not produce normally distributed data due to a high level of variation within several different outliers. This can be seen in Table 2, in which the median of the pre-test has a rather big difference between minimum and maximum values with the week-6 measurement.

Another big difference can also be seen in the measurement of BGL in week 4. This can be associated with the incidence of hyperglycemia that occurred in a patient in week 4 and 2 patients in week 6. The hyperglycemia incident in week 4 was a transient hyperglycemia. Meanwhile the other two incidences that occurred in week 6 were septic shock with hyperglycemia.

In our research, only 1/57 (1.7%) subjects had transient hyperglycemia, while Baillargeon et al., (2005) had 11% had transient hyperglycemia. The difference in the average age of the patients may be the reason. Baillargeon et al., (2005) reported that their patients were between 13-18 years of age. Pui et al., (1981) found that children aged 10 years old or older were more susceptible to hyperglycemia, due to an increase in insulin resistance associated with gonadotropic steroid hormone in puberty. In our research, only 28.1% of subjects were above 10 years of age.

None of the patients in our research had diabetic ketoacidosis (DKA). This result is in line with that of Robertson et al., (2008), whose study only had 6 out of 797 patients (0.8%) with DKA, even though they reported a high incidence of hyperglycemia. In our research, there were 2 patients who had hyperglycemia in week 6 with sepsis who then passed away due to septic shock.

Secondary hyperglycemia due to acute stress, which increases the production of glucose and insulin resistance, is not unheard of. However, this research did not find any statistically significant correlations between infection and hyperglycemia. This result is different compared to Sonabend et al., (2008), who reported that patients with hyperglycemia had an increased risk of bacteremia/fungemia up to 4.2 fold. This can be caused by a difference in follow-up time, where Sonabend’s research was retrospective and observed for any infection for one year. Another plausible cause is the different characteristics of the subjects, where other contributing factors such as obesity and older age, were scarce in our subjects.

A study conducted by Dare et al., (2013) found correlations between hyperglycemia, risk of infection, and early deaths during the induction phase of ALL. However, the incidence of hyperglycemia was also high (36%). The results obtained from our research, although statistically insignificant, were clinically relevant, which is reflected by the two severe infection cases that arose during our research.

We found no correlation between ALL stratification and the incidence of hyperglycemia in week 4 (p = 0.614) and week 6 (p = 0.373). There were also no significant correlations between the incidence of hyperglycemia (see Table 4) and other factors such as family history of diabetes mellitus, leukocyte count, nutritional status, CNS infiltration, and infection incidence. When the mean value of BGL was created after transforming the data into inversed BGL to make a normal data distribution, no difference was found between the mean value of BGL in pre and post-test for everyweek (Table 5). This shows that administering the steroid and L-asparaginase combination did not increase the mean value of BGL during the induction phase of ALL. Additionally, we analyzed the medial value of BGL pre and post-test (see Table 6) in order to see the difference in administering different kinds of corticosteroid (dexamethasone vs prednisone) towards BGL and due to the abnormal distribution. There was a significant difference (p = 0.038) in week 6. After, a test for the delta value in pre- and post-measurement was conducted to know the different effects in giving different kinds of corticosteroid (dexamethasone vs prednisone) towards the BGL in each week. From the relative risk count based on the mean value of the 3rd delta, it was shown that giving dexamethasone increased the likelihood of an above-average in the mean value of BGL up to 10.68 fold compared to prednisone. This result is also statistically significant with a confidence level of 1.528-74.733. Our finding shows that giving dexamethasone in combination with L-asparaginase increases the risk of changes in BGL compared to the mean value of BGL post-chemotherapy week 6 (Table 7). The relatively low prevalence of ALL and the small incidence of hyperglycemia in this research compared to other studies made it difficult to determine the effect of every factor, thus giving various results in several analysis.

Our research has some limitations. The small incidence of hyperglycemia (5.2%) in this research makes it difficult to determine the effect of risk factors on hyperglycemia. Another limitation is the presence of participant bias - patients were informed of the objectives and advantages of this research beforehand, which may have affected their diet during the induction phase of chemotherapy. Different laboratory standards among hospitals, such as measurement tools for BGL may also influence the quality of the data obtained, which should be evaluated later.
